# Enhanced charge separation and increased oxygen vacancies of h-BN/OV-BiOCl for improved visible-light photocatalytic performance†

**DOI:** 10.1039/c9ra01639b

**Published:** 2019-05-07

**Authors:** Wenhui He, Yawen Wang, Caimei Fan, Yunfang Wang, Xiaochao Zhang, Jianxin Liu, Rui Li

**Affiliations:** College of Chemistry and Chemical Engineering, Taiyuan University of Technology Taiyuan 030024 China wangyawen@tyut.edu.cn 1607232396@qq.com

## Abstract

The introduction of oxygen vacancies (OVs) on the surface of photocatalysts has already been proven to be an effective way to extend the light response to visible light and trap charge carriers, thereby promoting the photocatalytic performance. In this study, h-BN/OV-BiOCl composites were prepared using hexagonal boron nitride (h-BN) to further improve the visible-light photocatalytic activity of oxygen-vacancy-enriched bismuth oxychloride (OV-BiOCl). The composition and morphology of these materials were investigated, and the photocatalytic performance experiments showed that the introduction of h-BN could significantly improve the visible-light photocatalytic activity of OV-BiOCl, which was 1.7 and 1.4 times that of pure OV-BiOCl for the degradation of rhodamine B (RhB) and bisphenol A (BPA) when the h-BN content was 5 wt%, respectively. The role of h-BN was comprehensively investigated, and the results revealed that the presence of negatively charged h-BN could improve the separation efficiency of photoinduced electrons (e^−^) and holes (h^+^) by promoting the migration of positively charged h^+^ to the surface of the photocatalyst, as expected. Moreover, the oxygen vacancies in OV-BiOCl were increased in the presence of h-BN; this favored the activation of more adsorbed O_2_ for the oxidation of pollutants. Finally, a probable mechanism was proposed for the improved photocatalytic activity of the h-BN/OV-BiOCl composites. This study provides an insight into the roles of h-BN in oxygen-vacancy-enriched photocatalysts.

## Abstract 2

h-BN/OV-BiOCl composites were prepared to improve the visible-light photocatalytic activity of OV-BiOCl.

## Graphical abstract

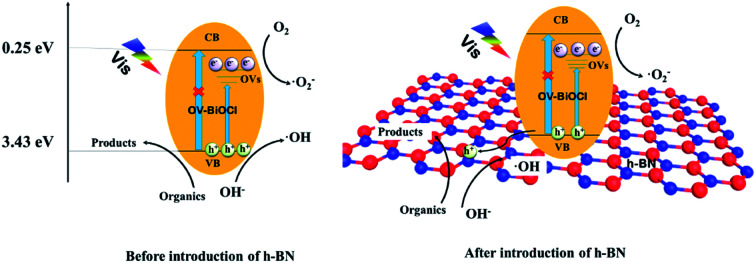

## 1. Introduction

The photocatalytic technology has attracted significant attention due to its wide application in the removal of pollutants and solar energy conversion.^1–3^ The design and development of highly efficient photocatalysts is the core issue in photocatalytic technology.^4^ Bismuth oxychloride (BiOCl), as a crucial kind of V–VI–VII ternary oxide semiconductor, has been proved to be a promising semiconductor photocatalyst for wastewater treatment because of its high photocatalytic activity and high chemical stability.^5–7^ However, the photocatalytic activity of pure BiOCl is still limited by its low response under visible light irradiation and high recombination rate of charge carriers. To solve these issues, many efforts have been proposed. For instance, specific structural design,^8,9^ crystal facet exposure,^10,11^ doping with metal or nonmetal ions^12,13^ and coupling with other materials^14–22^ have been developed to improve the photocatalytic activity of BiOCl. In addition to these, the formation of oxygen vacancies in BiOCl has been found to be an effective way to broaden the range of light absorption to the visible light region,^23–26^ and OV-rich BiOCl-based photocatalysts have shown more enhanced photocatalytic performance.^27–30^ However, the high rate of carrier recombination still limits their photocatalytic efficiency. Many researchers have combined OV-BiOCl with Au, Ag, Bi or g-C_3_N_4_ to increase the separation efficiency of photoinduced e^−^ and h^+^ through the acceleration of the electron transfer.^28,31–33^ However, considering that the recombination time of surface h^+^ (about 10 ns) is much shorter than that of e^−^ (about 100 ns), it would be more significant to promote the transfer of h^+^ for the enhancement of the photocatalytic performance.^34^ However, to date, no study has been reported on the improvement of the separation efficiency of visible-light-responding OV-BiOCl by the transfer of holes.

h-BN is a material similar to graphene and has good performance from its graphene-like structure, and several reports have been published about the potential application of h-BN in the catalytic field.^35,36^ In addition, when bulk h-BN is exfoliated to few-layer, the h-BN can be electronegative.^37^ If combining electronegative h-BN with other photocatalysts, it will attract photoexcited holes by electrostatic interaction and increase the charge separation, which has been proved by combinations of In_2_S_3_, gC_3_N_4_, and Bi_2_WO_6_.^37–39^ Inspired by the aforementioned concepts, it would probably also work well by combining with h-BN to improve the photocatalytic activity of OV-BiOCl.

In this work, h-BN/OV-BiOCl composites were successfully prepared by a solvothermal method. These as-prepared samples were investigated in detail in terms of their structures, morphologies, and photoelectric characterization. The photocatalytic activity evaluation was performed by assessing the degradation of the cationic dye RhB and colorless anionic BPA under visible light irradiation, and it was demonstrated that h-BN, as expected, could indeed improve the separating efficiency of e^−^ and h^+^ by promoting the migration of positive-charged h^+^ to the surface of the photocatalyst due to electrostatic interactions. Except for that, interestingly, it was found that the oxygen vacancies in OV-BiOCl were also increased with the presence of h-BN, which favored more ˙O_2_^−^ being produced to oxidize pollutants.

## 2. Experimental

### 2.1 Materials

All the chemicals were of analytical grade and were used as purchased from Aladdin (Shanghai, China) without further purification.

### 2.2 Catalysts preparation

#### 2.2.1 Preparation of h-BN/OV-BiOCl composites

The h-BN/OV-BiOCl composites were synthesized *via* a facile solvothermal process. First, 2 mmol Bi(NO_3_)_3_·5H_2_O and 0.8 g PVP were dissolved in 60 mL 0.1 mol L^−1^ mannitol solution with magnetic stirring for 30 min. Then, a certain amount of h-BN was added into the above solution with the help of ultrasonic treatment for 30 min. Then, 20 mL saturated sodium chloride solution was dropwise added into the above mixture under stirring. After being stirred for 60 min, a uniform white suspension was formed. Then, the suspension was transferred into a Teflon-lined 100 mL capacity autoclave, which was heated at 160 °C for 5 h and the reactor was then allowed to cool to room temperature naturally. The resulting samples were collected and washed with deionized water and ethanol four times and then dried at 60 °C in air for 24 h. The added contents of h-BN in the h-BN/OV-BiOCl composites were 0, 3, 5, and 8 wt%, respectively, and the corresponding obtained materials were denoted as OV-BiOCl, 3 wt% h-BN/OV-BiOCl, 5 wt% h-BN/OV-BiOCl, and 8 wt% h-BN/OV-BiOCl. For comparison, oxygen vacancy-poor BiOCl was prepared under the same conditions as used for OV-BiOCl without adding PVP, and denoted as OV-poor BiOCl.

#### 2.2.2 Preparation of h-BN

h-BN was prepared *via* calcination using boric acid and urea as the raw materials according to Rao *et al.*'s method^40^ Here, boric acid and urea at a mole ratio of 1 : 24 were dissolved in 40 mL distilled water, then the resulting solution was stirred and heated at 80 °C to obtain a white precursor. This white precursor was then calcined at 900 °C for 5 h under a nitrogen atmosphere to get the h-BN product.

### 2.3 Characterizations

X-ray powder diffraction (XRD) patterns of the as-synthesized samples were recorded on a D/max-2500 diffractometer with monochromatized Cu Kα radiation (*k* = 1.5406 Å) and the scanning range was 10° to 80° at a scan rate of 8° min^−1^. Fourier transform infrared (FT-IR) spectroscopy was performed on a Shimadzu 8400 spectrophotometer in the range 400–4000 cm^−1^ using KBr disk as the reference. X-ray photoelectron spectroscopy (XPS) was performed using a VG MultiLab 2000 system with a Mg Kα source operating at 20 kV. Scanning electron microscopy (SEM) images were obtained on a Nanosem 430 field emission scanning electron microscope with energy dispersive spectroscopy (EDS). Transmission electron microscopy (TEM) and high-resolution transmission electron microscopy (HRTEM) images were obtained using a JEOL-2011 microscope (Japan) at an acceleration voltage of 200 kV. UV-vis diffuse reflectance spectroscopy (DRS) was detected by a UV-2450 spectrophotometer in the range of 200–800 nm, with BaSO_4_ used as the standard. The photoluminescence (PL) spectra were acquired at room temperature using a Varian Cary Eclipse spectrometer, and the samples were excited at a wavelength of 319 nm. Room temperature electron spin resonance (ESR) under visible light irradiation and low-temperature electron paramagnetic resonance (EPR) spectra were recorded on a Bruker model JES-FA200 spectroscope. Moreover, the illumination time for the ESR was 5 min, with 5,5-dimethyl-1-pyrroline-*N*-oxide (DMPO) used as the spin trap in water and methanol, respectively. The zeta-potentials were acquired on a Malvern ZS90 with the samples dispersed in water.

### 2.4 Photocatalytic activity tests

The photocatalytic activities of the OV-BiOCl and h-BN/OV-BiOCl composites were evaluated by degrading RhB and BPA solution under visible light irradiation. A 350 W Xe lamp with a 420 nm filter plate was used as the visible light source. In detail, 0.02 g and 0.05 g of the photocatalysts were added in RhB solution (100 mL, 10 mg L^−1^) and BPA solution (100 mL, 10 mg L^−1^) respectively to produce a suspension at room temperature with magnetic stirring. Prior to turning on the light, the suspensions were kept stirring in the dark for 20 min to establish an absorption–desorption equilibrium between the photocatalyst and pollutant. At given irradiation intervals, 3 mL of the suspension was collected and centrifuged for further analysis of the concentration of the pollutants using a Varian Cary 50 probe UV-vis spectrometer. In order to detect the active species produced during the photocatalytic reaction process, the experiments for the free radicals of the superoxide radicals (˙O_2_^−^), holes (h^+^), and hydroxyl radicals (˙OH) capture were carried out by adding 0.02 g benzoquinone, 0.02 g ammonium oxalate, and 2 mL *t*-butanol in 100 mL 10 mg L^−1^ RhB solution, respectively. The degradation efficiency (%) was calculated according to the equation below:Degradation efficiency (%) = (*C*_0_ − *C*)/*C*_0_ × 100%where *C*_0_ and *C* are the initial adsorption equilibrium concentration and the concentration of the dye solution after *t* (min) of irradiation, respectively.

### 2.5 Photoelectrochemical measurements

The transient photocurrent responses and Mott–Schottky plots were measured on an electrochemical analyzer (CHI 660B Chenhua Instrument Company) in a standard three-electrode system. The counter electrode was a platinum wire and the reference electrode was a saturated Ag/AgCl electrode. The electrolyte solution was Na_2_SO_4_ aqueous solution (1 M). A 500 W Xe arc lamp with an ultraviolet filter was used as the visible-light source. Mott–Schottky plots were obtained at a frequency of 1 kHz.

## 3. Results and discussion

### 3.1 Compositional and structural information

**Fig. 1:**
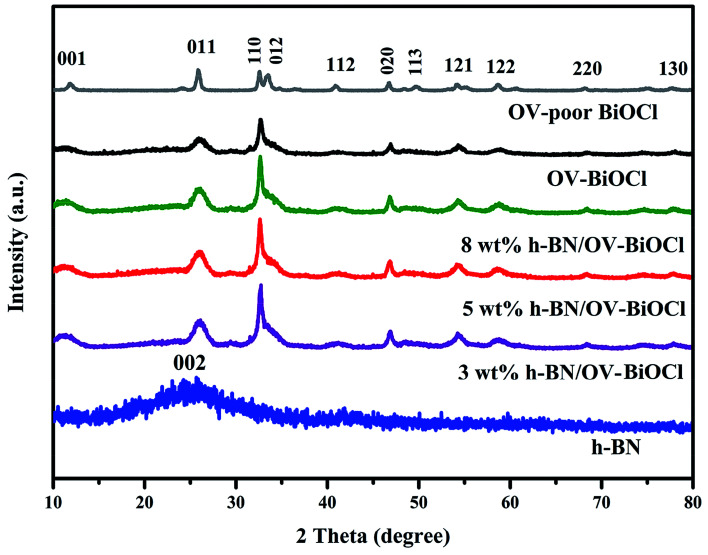
XRD patterns of the as-prepared h-BN, OV-poor BiOCl, OV-BiOCl, and h-BN/OV-BiOCl composites.

The composition and phase structures of the samples were confirmed by XRD analysis. As shown in Fig. 1, the XRD pattern of h-BN displayed broadened peaks located at around 25.5, which could be ascribed to the (002) plane of BN (JCPDS no. 34-0421). The characteristic diffraction peaks of OV-poor BiOCl and OV-BiOCl were in good agreement with the BiOCl (JCPDS card no. 73-2060). The as-synthesized h-BN/OV-BiOCl composites with different weight ratios of h-BN exhibited similar XRD patterns to OV-BiOCl, and there was no shift of the characteristic diffraction peaks, indicating that the h-BN only existed on the surface of OV-BiOCl and it did not change the lattice structure of OV-BiOCl. In addition, in the case of the h-BN/OV-BiOCl composites, no diffraction peak of h-BN could be observed, which may be due to high dispersion, limited amount, and low diffraction intensity of h-BN in the h-BN/OV-BiOCl composites.^41,42^

**Fig. 2:**
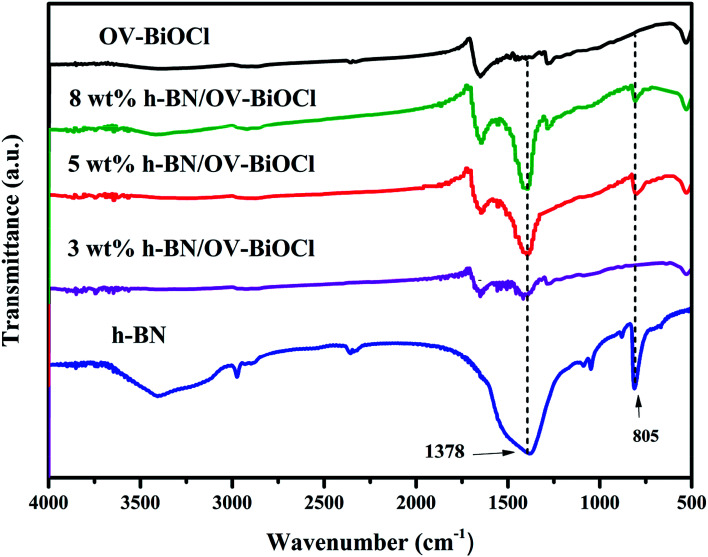
FTIR spectra of h-BN, OV-BiOCl, and h-BN/OV-BiOCl composites.

The compositions of the h-BN, OV-BiOCl, and h-BN/OV-BiOCl composites were confirmed by FT-IR analysis, as shown in Fig. 2. The characteristic peaks at 1378 and 805 cm^−1^ for pure h-BN could be attributed to the stretching vibration of the B–N bond and B–N–B out-of-plane, respectively. The absorption peak at 516 cm^−1^ for OV-BiOCl was attributed to the Bi–O stretching mode. In the case of the h-BN/OV-BiOCl composites, the characteristic absorption peaks of the Bi–O stretching mode, and B–N and B–N–B stretching vibrations could all be observed, suggesting that h-BN had been successfully introduced into the OV-BiOCl.

**Fig. 3:**
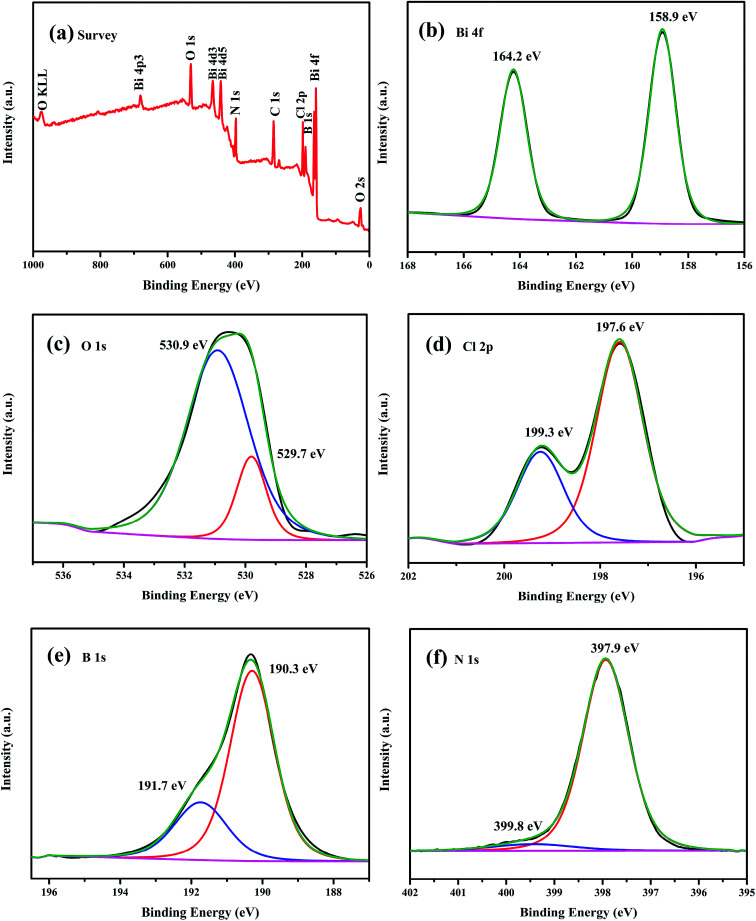
XPS spectra of the as-prepared 5 wt% h-BN/OV-BiOCl composite: (a) survey spectra, (b) Bi 4f, (c) O 1s, (d) Cl 2p, (e) B 1s, and (f) N 1s.

In order to elucidate the element composition and valence states of the as-obtained composite, the chemical state of the as-prepared 5 wt% h-BN/OV-BiOCl was carefully investigated by XPS. The survey scan spectrum (Fig. 3a) showed that Bi, O, Cl, B, and N elements were present in the h-BN/OV-BiOCl composites. As shown from the high-resolution image of Bi 4f (Fig. 3b), two strong characteristic peaks at about 164.2 and 158.9 eV could be assigned to Bi 4f5/2 and Bi 4f7/2, which were ascribed to Bi ions present in a valence of +3 in the composites. The peaks at 530.9 and 529.7 eV of the O 1s spectrum were assigned to surface OH species and Bi–O–Bi bonds in the composites (Fig. 3c). The Cl 2p region consisted of two major peaks located at 199.3 and 197.6 eV, corresponding to the binding energies of Cl 2p1/2 and Cl 2p3/2 of Cl^−^, respectively (Fig. 3d). Fig. 3e and f show the high-resolution spectra of B 1s and N 1s. The peaks of B in 5 wt% h-BN/OV-BiOCl were located at 191.7 and 190.3 eV, respectively, and were attributed to B–O bonds formed by B-site hydroxylation and B–N bonds respectively, while the peaks of N 1s at 399.8 and 397.9 eV were assigned to nitrogen species on the surface (adsorbed NO_*X*_, NO^2−^, N_2_, NH_*X*_)^43^ and N^3−^ in BN, respectively, indicating that h-BN has combined with OV-BiOCl successfully.

### 3.2 Morphology and microstructure analysis

**Fig. 4:**
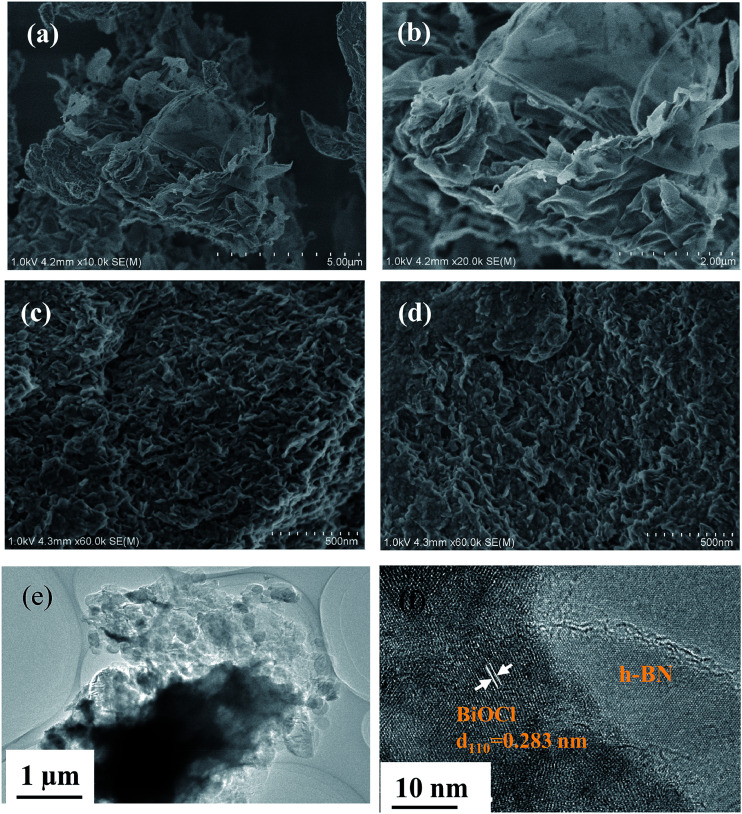
SEM images of (a and b) h-BN, (c) OV-BiOCl, and (d) 5 wt% h-BN/OV-BiOCl, respectively. (e) TEM image and (f) HETEM image of 5 wt% h-BN/OV-BiOCl.

The morphology and microstructure of the as-prepared h-BN, OV-BiOCl, and h-BN/OV-BiOCl composites were observed by assessing their SEM and TEM images. As seen in Fig. 4a and 4b, the local morphology of the h-BN prepared by calcination looks very thin, indicating h-BN is few-layer. From Fig. 4c and d, it can be seen that the as-prepared OV-BiOCl (Fig. 4c) exhibits aggregated, sheet-like stacking layers. The morphology of 5 wt% h-BN/OV-BiOCl (Fig. 4d) looks no different from OV-BiOCl, but the EDS element mapping shows the elements Bi, Cl, O, B, and N were uniformly distributed in the 5 wt% h-BN/OV-BiOCl composite (Fig. S1†). TEM analysis was applied to explore the microstructure of the h-BN/OV-BiOCl composites. From the TEM image (Fig. 4e) of the 5 wt% h-BN/OV-BiOCl composite, it can be observed that the OV-BiOCl particles are located on the surface or edge of h-BN. The sufficient contact region between h-BN and OV-BiOCl may make a great contribution to efficient photocatalytic-induced carrier transfer. HRTEM (Fig. 4f) was employed to prove the formation of a heterojunction between h-BN and OV-BiOCl. The image shows a clear lattice fringe set with *d*-spacings of 0.27 nm, corresponding to the (110) lattice plane of BiOCl. The above results further demonstrate that the h-BN was modified on OV-BiOCl to construct the h-BN/OV-BiOCl composites.

### 3.3 Pollutant photodegradation and photoelectric properties

**Fig. 5:**
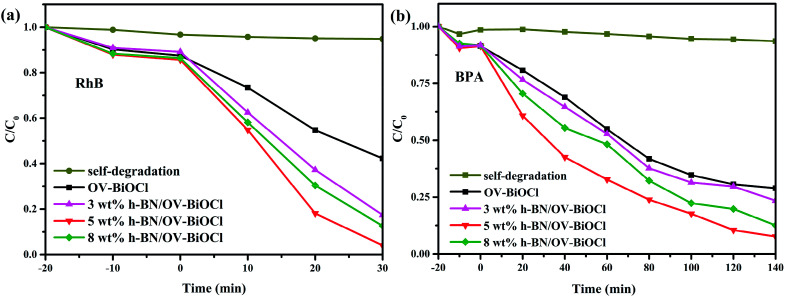
Photocatalytic degradation of (a) RhB and (b) BPA in the presence of the samples under visible-light irradiation.

The photocatalytic performance tests of the OV-BiOCl and h-BN/OV-BiOCl composites were carried out employing the cationic dye RhB and the colorless anionic BPA as the target pollutants under visible light irradiation, and the results are shown in Fig. 5a and 5b, respectively. It can be seen that the photodegradations of RhB and BPA displayed a similar trend. Only a little bit of RhB and BPA was removed without any photocatalyst, which suggests that the self-degradation of RhB and BPA can be ignored. About 58% RhB could be photodegraded in 30 min irradiation and 72% BPA in 140 min by OV-BiOCl. Pure h-BN showed an excellent adsorption performance but negative photocatalytic activity to RhB and BPA, as shown in Fig. S2.† This is probably because the content of h-BN was not much; therefore, the adsorption performance of the h-BN/OV-BiOCl composites did not display much difference compared to OV-BiOCl. While the photocatalytic activity of OV-BiOCl was significantly enhanced with the introduction of h-BN. Notably, the 5 wt% h-BN/OV-BiOCl composite exhibited the highest photocatalytic activities of 96% and 93% for RhB and BPA removal, respectively. When the content of h-BN exceeded 5 wt%, the photocatalytic activity of the as-prepared sample decreased. Therefore, a certain amount h-BN loading is conducive to charge migration from OV-BiOCl to h-BN, while too high a loading content of h-BN may limit the number of surface active sites of OV-BiOCl, leading to a decline in the photocatalytic activity.^41^ After four cycles, there was no apparent deactivation for the photocatalysts, as shown in Fig. S4,† indicating the catalysts had high photostability.

**Fig. 6:**
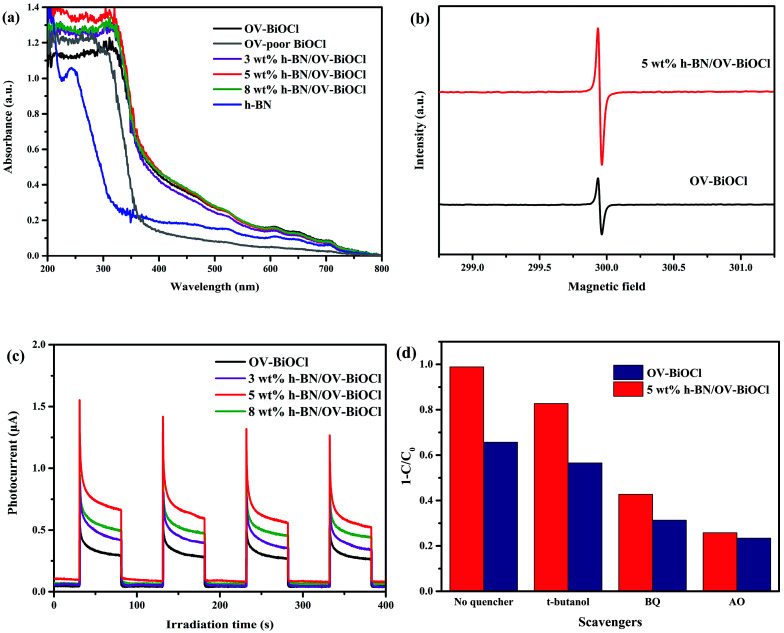
(a) UV-vis diffuse reflectance spectra of h-BN, OV-poor BiOCl, OV-BiOCl, and h-BN/OV-BiOCl composites. (b) EPR spectra of OV-BiOCl and 5 wt% h-BN/OV-BiOCl collected at low temperature. (c) Transient photocurrent response of OV-BiOCl and h-BN/OV-BiOCl composites. (d) Scavenger experiments of reactive species during the photocatalytic degradation of RhB under visible-light irradiation.

For better understanding the reason for the enhanced photocatalytic activity of h-BN/OV-BiOCl, the optical absorption properties of the as-prepared samples were investigated by UV-vis DRS. As revealed in Fig. 6a, compared to the OV-poor BiOCl, the light absorbance of OV-BiOCl exhibited a significant red-shift and a distinct absorption tail, which could be attributed to the existence of oxygen vacancies.^44^ The oxygen vacancies should be formed *via* a solvothermal route assisted with PVP in the preparation process.^45^ After introducing h-BN, the absorbing edge and light-harvesting ability of OV-BiOCl only showed a slight change, and the band-gap energies (*E*_g_) of the catalyst were estimated to be 3.34, 3.18, 3.17, 3.11, and 3.14 eV for OV-poor BiOCl, OV-BiOCl, 3 wt% h-BN/OV-BiOCl, 5 wt% h-BN/OV-BiOCl, and 8 wt% h-BN/OV-BiOCl, respectively (the calculation process is shown in the ESI†), which revealed the ability to harvest light should not be the main factor to realizing the enhanced photocatalytic activity of h-BN/OV-BiOCl composites.

The presence of oxygen vacancies in the samples was further confirmed by EPR at low temperature. As shown in Fig. 6b, the remarkable EPR signal at *g* = 2.001 is the typical characteristic signal of oxygen vacancies. The oxygen vacancies signal for 5 wt% h-BN/OV-BiOCl was stronger than that of OV-BiOCl, indicating that the addition of h-BN can increase the OV concentrations of OV-BiOCl. During preparation, the negative-charged h-BN may prefer to adsorb on the surface of Bi^3+^ and occupy a number of positions that the O atom should usually be in, giving rise to the increase in OVs.

The transient photocurrent response was also measured and the results are exhibited in Fig. 6c. The prompt photocurrent response of the as-prepared materials corresponded well with on/off cycles of visible-light irradiation. It could be seen that the photocurrent intensity of h-BN/OV-BiOCl electrodes were higher in comparison to the OV-BiOCl electrode. The photocurrent is mainly produced by the diffusion of photoexcited carriers. According to the DRS results, the presence of h-BN had no apparent influence on the light harvesting of OV-BiOCl. Therefore, the enhanced photocurrent response for the h-BN/OV-BiOCl means that the photogenerated electron–hole pairs are separated more effectively, which was also confirmed by the PL analysis (Fig. S3†). For 5 wt% h-BN/OV-BiOCl, the emission intensity decreased significantly when compared with the OV-BiOCl at a similar emission position, which indicates that the introduction of h-BN can increase the separation efficiency of the electron–hole pairs of OV-BiOCl.

To explore the active radicals of the h-BN/OV-BiOCl composites, we performed free-radical-trapping experiments in the catalytic systems of OV-BiOCl and 5 wt% h-BN/OV-BiOCl over RhB aqueous solutions under visible-light irradiation by adding different scavengers (ammonium oxalate, *t*-butanol, and benzoquinone were used to trap h^+^, ˙OH, and ˙O_2_^−^, respectively). As shown in Fig. 6d, when *t*-butanol was added, the photodegradation efficiency declined by 9% and 13% for the OV-BiOCl and 5 wt% h-BN/OV-BiOCl, respectively, implying that the ˙OH was slightly responsible for the photodegradation. It could be observed that benzoquinone (34% and 56% depression for OV-BiOCl and 5 wt% h-BN/OV-BiOCl, respectively), and especially ammonium oxalate (42% and 73% depression for OV-BiOCl and 5 wt% h-BN/OV-BiOCl, respectively) had obvious influences on the photocatalytic activity. Depending on the results, it could be confirmed that h^+^ plays a crucial role and ˙O_2_^−^ also plays an important role in the photocatalytic degradation processes for h-BN/OV-BiOCl.

**Fig. 7:**
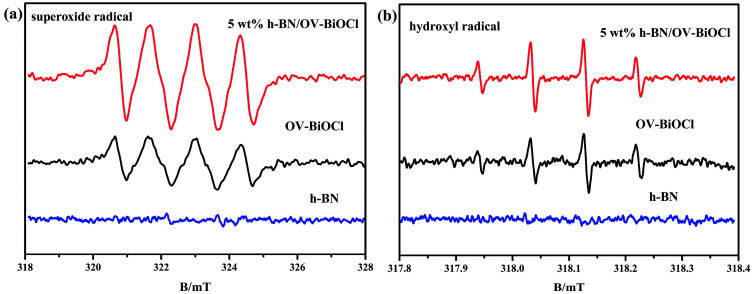
ESR spectra of radical adducts trapped by DMPO in h-BN, OV-BiOCl, and h-BN/OV-BiOCl composites: (a) methanol dispersion and (b) aqueous dispersion under visible-light irradiation.

We also used ESR spin-trap detection with DMPO to measure the signals of ˙O_2_^−^ and ˙OH of h-BN, OV-BiOCl, and h-BN/OV-BiOCl under visible-light irradiation, and the results are presented in Fig. 7. For h-BN, there were no significant ˙O_2_^−^ and ˙OH signals appearing, while both ˙O_2_^−^ and ˙OH appeared with OV-BiOCl and h-BN/OV-BiOCl. According to the DRS results, the *E*_VB_ and *E*_CB_ of OV-BiOCl were estimated to be 3.43 and 0.25 eV, respectively (the calculation process is shown in the ESI†). Because the reduction potentials of ˙OH/OH^−^ and O_2_/˙O_2_^−^ were 2.38 and −0.046 eV, the oxidation of OH^−^ to ˙OH could be achieved, while the reduction of O_2_ to ˙O_2_^−^ was not feasible. However, when OVs were present in BiOCl, the surface OVs with typical defect states can not only improve the absorption in the visible-light region, but can also act as active sites to activate O_2_ by e^−^.^46,47^ Obviously, the intensity of ˙O_2_^−^ peaks over h-BN/OV-BiOCl was much stronger than that for OV-BiOCl, implying the activation of molecular oxygen was greatly enhanced in h-BN/OV-BiOCl. This may be due to the increase in OVs in BiOCl and the increased separation efficiency of the photoinduced e^−^ and h^+^ after combining with h-BN, which causes more ˙O_2_^−^ radicals produced by capturing e^−^ to adsorb O_2_ at the OVs.^31^ In Fig. 7b, despite ˙OH not being the main radical in degradation of the pollutant in this case, the intensity of ˙OH also increased after combining with h-BN on OV-BiOCl, which indicates the separation efficiency of photoinduced e^−^ and h^+^ was promoted by the presence of h-BN.

**Fig. 8:**
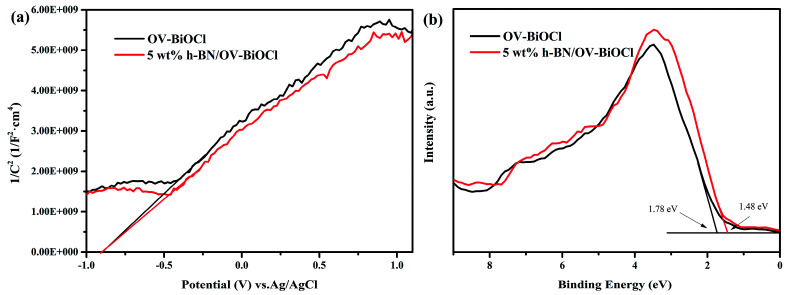
(a) Mott–Schottky analysis of OV-BiOCl and 5wt% h-BN/OV-BiOCl electrodes at a frequency of 1 kHz in the dark, (b) valence-band XPS spectra of OV-BiOCl and the 5 wt% h-BN/OV-BiOCl sample.

In order to further detect the impact of h-BN on how to promote the separation efficiency of the photoinduced e^−^ and h^+^, we performed Mott–Schottky plots and valence-band XPS to estimate the flat band level and valence band level of OV-BiOCl and 5 wt% h-BN/OV-BiOCl approximately, as shown in Fig. 8. It is well known the flat band potential is strongly related to the bottom of the conduction band. Fig. 8a shows that the flat band value of 5 wt% h-BN/OV-BiOCl was the same as for the OV-BiOCl, which implied that the position of photogenerated electrons in the h-BN/OV-BiOCl composites was similar to OV-BiOCl. However, Fig. 8b indicates that the valence band of 5 wt% h-BN/OV-BiOCl (about 1.48 eV) is different from that of OV-BiOCl (about 1.78 eV), which indicates the position of photogenerated holes in the h-BN/OV-BiOCl composites was not in accordance with in OV-BiOCl. These results fully prove that the presence of h-BN tends to affect the photoinduced holes instead of electrons. The zeta-potential of h-BN was tested, and the result was about −32 mA, which is in accordance with the previous literature.^48,49^ These suggest the negative-charged h-BN modified on the OV-BiOCl surface can attract the positive-charged photoinduced holes to the surface of the photocatalyst due to electrostatic interactions, which accordingly will result in an enhancement of the separation efficiency of e^−^ and h^+^, with more holes available to accelerate the oxidation of pollutants. The phenomenon where h^+^ plays a more crucial role for h-BN/OV-BiOCl than for OV-BiOCl (Fig. 6d) also confirmed this conclusion. This situation is similar to that of surface anionized TiO_2_ by phosphate, fluoride, and sulfate, where the anion-derived surface negative charges are able to draw the photogenerated holes to the TiO_2_ surface by electrostatic force. Thus, more holes are available at the interface, which remarkably accelerates the oxidation of surface adsorbate.^50^

### 3.4 Possible photocatalytic mechanism

**Fig. 9:**
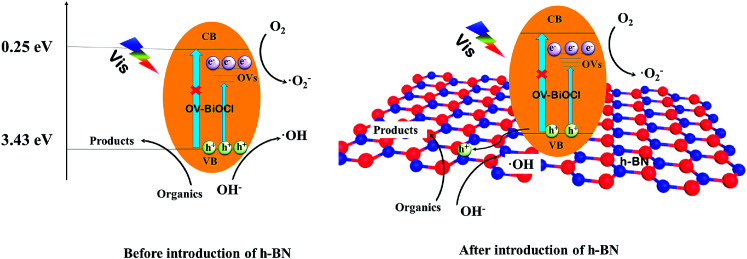
The possible photocatalytic mechanism of OV-BiOCl and the h-BN/OV-BiOCl composites.

Based on the above analysis, a possible photocatalytic mechanism for the h-BN/OV-BiOCl composites is proposed. As presented in Fig. 9, when OV-BiOCl is irradiated by visible light, the electrons on the valence band are excited to the oxygen vacancies states, leaving holes on the valence band. Owing to the special property of OVs in BiOCl, separated electrons can react with adsorbed O_2_ to form ˙O_2_^−^ at OVs.^46,47^ In this case, most of the separated holes can oxidize the pollutant directly and a small number of holes can react with surface adsorbed OH^−^ or H_2_O to form ˙OH. With the h^+^, ˙O_2_^−^, and ˙OH, organic pollutants can be degraded. When h-BN is introduced, the negatively charged h-BN on OV-BiOCl facilitates the migration of h^+^ to the surface of h-BN, which can improve the separation efficiency of e^−^ and h^+^; thereby, inducing more h^+^ to migrate to oxidize the pollutants directly. Interestingly, the oxygen vacancies are also increased with the presence of h-BN, accompanied with the increased separating efficiency of e^−^ and h^+^, favoring more ˙O_2_^−^ produced by absorbing more oxygen molecules to react with more separated e^−^. As a result, the improved separation efficiency of e^−^ and h^+^ and increased ˙O_2_^−^ synergistically result in the enhanced photocatalytic performance of h-BN/OV-BiOCl.

## 4. Conclusions

In conclusion, we successfully prepared h-BN/OV-BiOCl composites by a solvothermal method. The XRD, FTIR, and HRTEM results showed that h-BN was modified on the surface of OV-BiOCl and had no influence on the lattice structure of OV-BiOCl. The DRS results showed h-BN had little impact on the optical property of OV-BiOCl, but could enhance the separation efficiency of the photogenerated charge carriers according to the results from the transient photocurrent response tests. The zeta-potential, Mott–Schottky plots, and valence-band XPS tests were used to detect in detail the role of h-BN, and it was found that the negative-charged h-BN facilitates the migration of h^+^ to the surface of the photocatalyst by electrostatic interactions, inducing enhanced charge separation. Interestingly, the OVs on OV-BiOCl could also be increased after the introduction of h-BN, resulting in more ˙O_2_^−^ being formed. As a result, the h-BN/OV-BiOCl composites displayed improved photocatalytic activities on RhB and BPA degradation under visible-light irradiation compared with OV-BiOCl.

## Conflicts of interest

There are no conflicts to declare.

## Supplementary Material

RA-009-C9RA01639B-s001
