# Supplementary material for: Enhanced charge separation and increased oxygen vacancies of h-BN/OV-BiOCl for improved visible-light photocatalytic performance
Source: RSC Adv. 2019 May 7;9(25):14286–95. doi: 10.1039/c9ra01639b (PMC9064020; doi:10.1039/c9ra01639b)
Supplement: RA-009-C9RA01639B-s001 [file RA-009-C9RA01639B-s001.pdf]

Electronic Supplementary Material (ESI) for RSC Advances.
This journal is © The Royal Society of Chemistry 2019

Supplementary Material

Enhanced charge separation and increased oxygen vacancies of h-BN/OV-BiOCl for improved visible light photocatalytic performance

Wenhui He[a], Yawen Wang[a*], Caimei Fan[a*], Yunfang Wang[a], Xiaochao Zhang[a], Jianxin Liu[a], Rui Li[a]

College of Chemistry and Chemical Engineering, Taiyuan University of Technology, Taiyuan 030024, China

Corresponding author:E-mail address: wangyawen@tyut.edu.cn (Dr. Yawen Wang), 1607232396@qq.com (Prof. Caimei Fan)

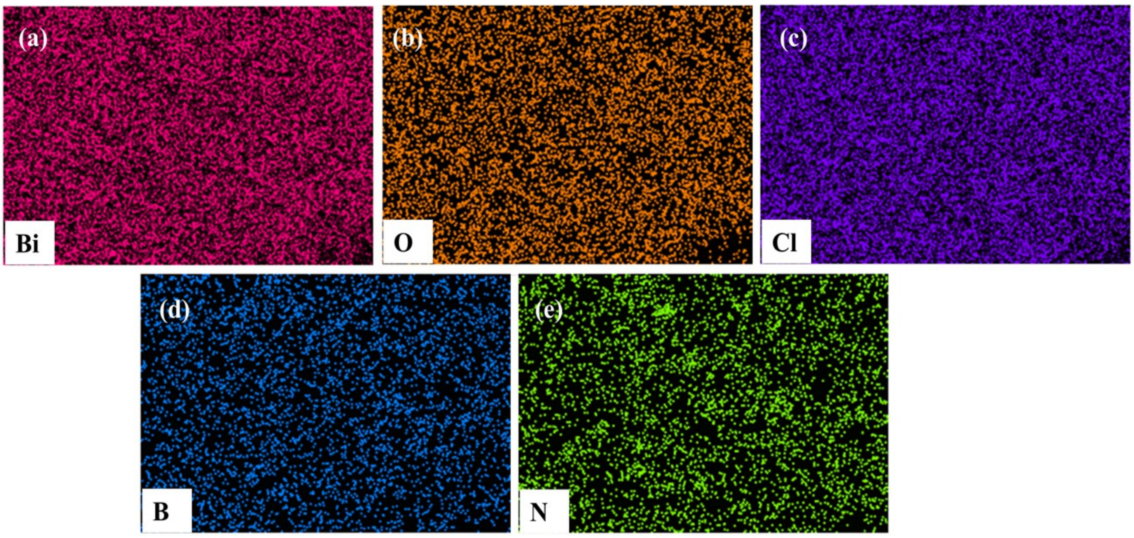

Fig. S1 (a)-(e) the EDS elemental mapping of Bi, O, Cl, B and N in 5wt% h-BN/OV-BiOCl composite.

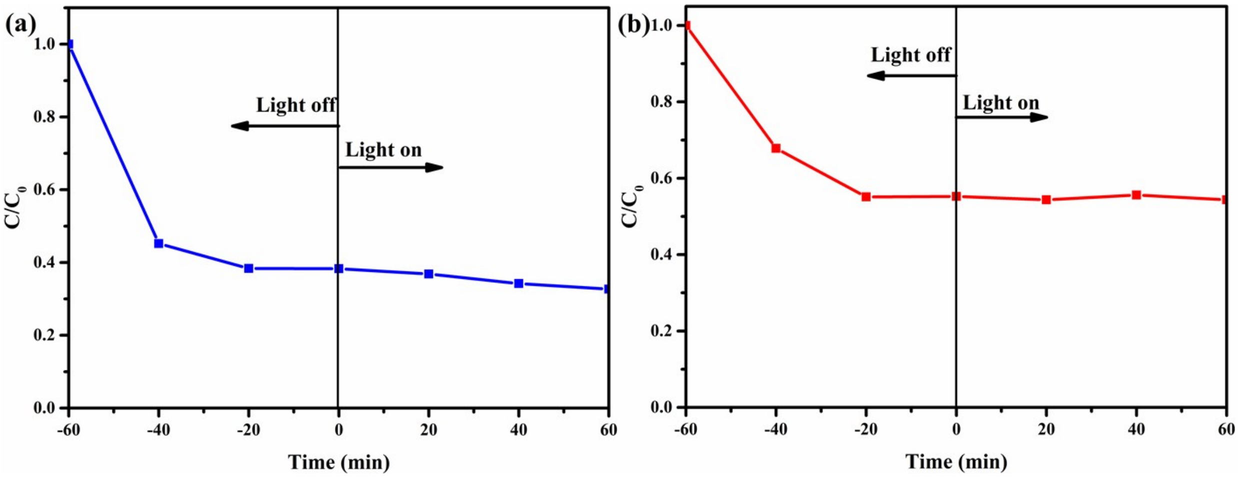

Fig. S2 Adsorption and photocatalytic degradation curves of h-BN for (a) RhB and (b) BPA under visible light irradiation. (0.02 g and 0.05 g of h-BN were added in a RhB solution (100 mL, 50 mg/L) and BPA solution (100 mL, 50 mg/L)).

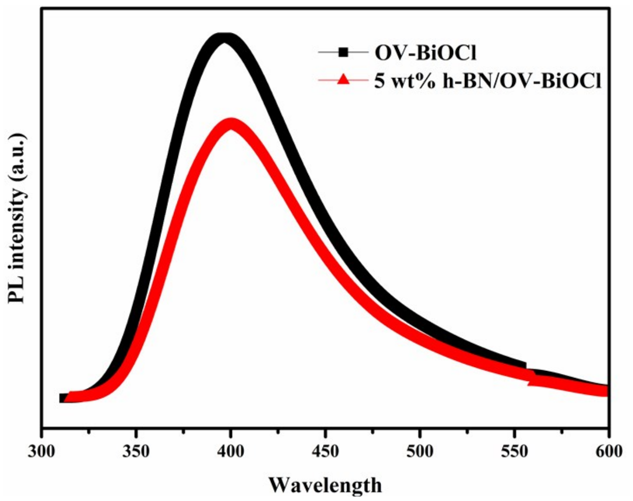

Fig. S3 PL of OV-BiOCl and 5 wt% h-BN/OV-BiOCl samples.

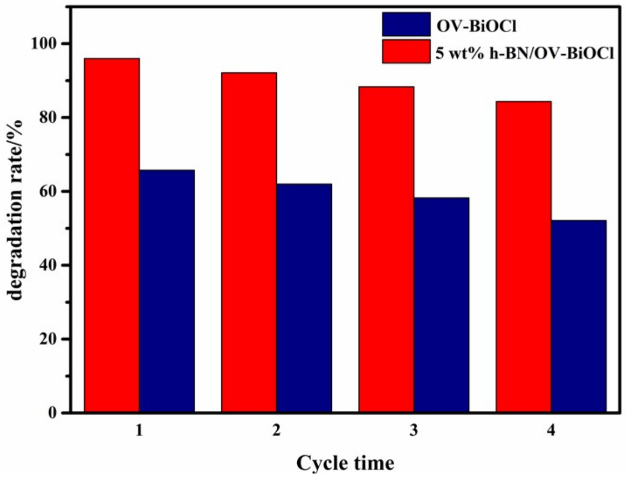

Fig. S4 Cycling runs of OV-BiOCl and 5 wt% h-BN/OV-BiOCl photocatalysts for RhB degradation.

Band-gap energy calculation process of OV-BiOCl and h-BN/OV-BiOCl

According to the formulas $Eg = 1240/\lambda$ (λ is wavelength value that extension of every line slope intersects with abscissa), λ is about 371, 380, 391, 399 and 395 nm, and Eg is calculated about 3.34, 3.18, 3.17, 3.11 and 3.14 eV for OV-poor BiOCl, OV-BiOCl, 3 wt% h-BN/OV-BiOCl, 5 wt% h-BN/OV-BiOCl and 8 wt% h-BN/OV-BiOCl, respectively.

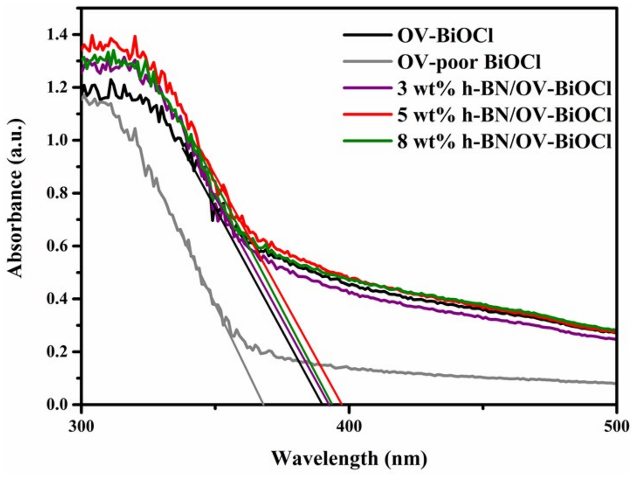

Fig. S5 UV-vis diffuse reflectance spectra of OV-BiOCl, OV-poor BiOCl and h-BN/OV-BiOCl composites.

Energy band calculation process of OV-BiOCl

According to the formulas E_{CB} = χ (AaBbCc)－1/2Eg＋E_0 and E_{VB} = E_{CB}＋Eg, where Eg is the semiconductor band gap, E_{CB} is the conduction band minimum value, E_{VB} is the valence band maximum value, E_0 is the free electron energy relative to the standard hydrogen electrode (-4.5 eV) , χ (AaBbCc) is the absolute electronegativity of the semiconductor AaBbCc calculated by the electronegativity geometric mean of the three elements, the value for BiOCl is calculated to be 6.34, and Eg is 3.18 eV acquired from DRS analysis. Moreover, the CB potential can be obtained by the formula E_{CB} = E_{VB} – Eg, the E_{VB} and E_{CB} of BiOCl are estimated 3.43 eV and 0.25 eV.
